# Health benefits of short Taichi Qigong exercise (STQE) to University Students’ core strength, lower limb explosive force, cardiopulmonary endurance, and anxiety: A Quasi experiment research

**DOI:** 10.1097/MD.0000000000037566

**Published:** 2024-03-29

**Authors:** Feng Wang, Syed Kamaruzaman Bin Syed Ali

**Affiliations:** a Department of Education Foundations and Humanities, Faculty of Education, University of Malaya, Kuala Lumpur, Malaysia.

**Keywords:** anxiety, cardiopulmonary endurance, core strength, health, lower limb explosive force, outcome, Taichi Qigong exercises

## Abstract

**Background:**

College students’ physical fitness has declined over the past decades. Taichi Qigong exercise offers numerous health benefits and could serve as a suitable option for them. Traditional programs, however, are time-consuming and necessitate long-term commitments. Therefore, a more cost-effective intervention is needed.

**Methods:**

The study enrolled a total of 31 students who actively participated in a 5-week STQE program, consisting of three 60-minute sessions per week. Physical and mental health assessments included the Plank test, vital capacity measurement, 1000/800 m run test, standing jump, and the Zung Self-Rating Scale. Data analysis was performed using SPSS.

**Results:**

Following the STQE intervention, participants showed improvement in core strength (28.1 seconds in the Plank test, *P* = .025) and lower limb explosive force (6.52 cm in the standing jump test, *P* = .011), accompanied by a decrease in anxiety levels (a reduction of 3.41 in the Zung Self-Rating Scale, *P* = .039). However, no significant improvements were observed in cardiopulmonary endurance, as evidenced by a non-significant increase of 237.84 mL in vital capacity (*P* = .134) and a non-significant reduction of 1.6 seconds in the 1000/800 m run test (*P* = .764).

**Conclusion:**

The study suggests that the STQE program effectively improves core strength, lower limb explosive force, and reduces anxiety levels among university students.

## 1. Introduction

There is an increasingly obvious trend of low physical activity levels among the youth population in China.^[1]^ Consistent with it, the physical fitness of Chinese college students has continuously decreased during the past 20 years.^[2]^ According to the Report on Nutrition and Chronic Disease Status of Chinese Residents in 2020, the mean body weight of Chinese young adults has increased by 3.4 kg (male) and 1.7 kg (female) since 2015.^[3]^ Moreover, a national observational survey also revealed that the prevalence of overweight rose from 12.82% in 2008 to 28.45% in 2018, and the prevalence of obesity increased from 1.62% in 2008 to 4.95% in 2018.^[4]^ Low physical activity levels, poor physical fitness, and obesity are the most common causes of reduced cerebral blood flow, premature death, along chronic non-communicable diseases for adolescents.^[5,6]^

Core strength is crucial for injury prevention, enhancing body stability, regulating the center of gravity, and transmitting strength between the upper and lower limbs.^[7]^ It involves a series of muscles (such as the transverse abdominis, multifidus, diaphragm, and pelvic floor muscles).^[8]^ Cardiopulmonary endurance is an individual’s ability to maintain long-term exercise or physical activity without tiring; it also refers to the heart’s and lungs’ ability to deliver oxygen to working muscles during continuous exercise.^[9,10]^ Lower limbs explosive force is characterizing muscular work in a short time and grants the largest acceleration to the organism via fast muscular effort.^[11–13]^ Anxiety represents the most common psychological disorder for young adults, it includes a series of negative disorders (phobia, panic, social anxiety, and so on).^[14,15]^ Therefore, all those variables are certain to be linked to individuals’ health and physical fitness.

Taichi and Qigong are 2 popular mind-body exercises all over the world,^[16,17]^ and previous studies have warranted that Taichi and Qigong have various benefits for practitioners, such as chronic heart failure,^[18]^ cancer,^[19,20]^ mental disorders^[21]^ and so on. The common period of Taichi and Qigong exercise usually ranges from 12 to 24 weeks.^[18,22,23]^ However, today is an increasingly fast-paced society,^[24,25]^ and residents may be unwilling to conduct such long-term exercise. Consequently, a short-term exercise program will be desired for them. Therefore, the short Taichi Qigong exercise program is very interesting to be studied.

The purpose of this study is to examine the effectiveness of a short Taichi Qigong exercise (STQE) on university students’ physical and mental health. The specific objective is to investigate the effectiveness of STQE on core strength, cardiopulmonary endurance, low limb explosive force, and anxiety for university students. Hypothesis 1, STQE has a significant improvement on university students’ core strength, cardiopulmonary endurance, and low limb explosive force. Hypothesis 2, STQE has a significant reducing effect on university students’ anxiety levels.

## 2. Material and methods

### 2.1. Research design

This research aims to examine the effectiveness of STQE on university students’ physical and mental health. A quasi-experimental pre and posttest design will be involved in the study. There is only one independent variable, and it is the STQE. Dependent variables are core strength, cardiopulmonary endurance, lower limbs explosive force, as well as anxiety level. At the beginning of the study, a pretest will be taken to record the baseline of dependent variables. Research will take a posttest to measure the outcomes of the 4 dependent variables. A mean value and a paired *t* test are used to assess the effectiveness. This trial was conducted between November 17 and December 30, 2020. The location is a public college in Nanning, in the south of China.

### 2.2. Ethical concern

This study obtained the ethical approval from the academic ethical board, Guangxi Vocational College of Technology and Business (No. 202005003). Besides, all involved volunteers have signed the consent form before join this research.

### 2.3. Participants and sampling and power

The population of this study is university students. An investigation revealed that university students were not physically active (only 7% of students reported having a very active lifestyle, and 4% had quite good nutritional knowledge).^[26]^ Physical activity is positive for resilience and emotional intelligence.^[27]^

The sampling method proposed in this study was used to select participants. Inclusion criteria are: free to attend the training and, no experience of Taichi and Qigong. The exclusion criteria are: having a serious health problem, missing 5 or more training sessions. According to the guidelines of Mazlan Ismail, at least 23 participants are needed in the intervention exercise experiment.^[28]^ To acquire better results, researchers enlarge the sample size. Finally, a total of 31 students (26 female, 5 male) from a public college were enrolled in this study. To validate the sample size, we utilized Gpower 3.1 software to calculate the study’s power. The statistical test chosen for family means comparison was the *t* test for 2 dependent means. Input parameters were set as follows: 1 tail, effect size (dz) = 0.5, β/α ratio = 1, and sample size = 31. The results revealed a power of 0.9, affirming the study’s robustness with a power of 0.9.

### 2.4. Short Taichi Qigong Exercise

The STQE is an original exercise program that was formulated by the researcher independently. It has 2 characteristics: first, it is a short period, which is the most important principle. Second, it combines Tai Chi and Qigong together. Specifically, STQE maintains a continuous period of 5 weeks, 3 times each week, and 60 minutes for each training. During the training session, begins with a 5-minute warm up, continues for 25 minutes of Taichi training, followed by 25 minutes of Qigong training, and ends with 5 minutes for a cool down. Taichi training is the latest form of Tai Chi, which is called Taiji Bafawubu.^[29]^ Qigong training is a static bent standing practice which is also known as Zhanzhuang.^[30]^ The details of STQE are available upon request to the first author (Feng Wang) of this study.

### 2.5. Measurements for DV

The plank test (PT) will be used to assess core strength. During this test, the individual has to maintain a prone position in which the body weight is supported by the toes and forearms.^[31]^ Longer time in the PT indicates better core strength for participants. The average means and standard deviation is 124 ± 72 and 83 ± 63 seconds for male and female, respectively.^[32]^ Cardiopulmonary endurance, it will be measured through 2 methods: the vital capacity (VC) and the 1000/800 m run test (RT). Students were instructed to perform maximal slow expiration via a mouthpiece connected to the spirometer during a VC test.^[33]^ The 1000/800 m run was assessed via the record time (accurate to minutes and seconds) to complete the distance, 1000 m for boys and 800 m for girls.^[33]^ Greater VC and shorter time in the RT represent better cardiopulmonary endurance (the cutoff of VC and 1000/800 m run is 3100 mL/2000 mL and 272 seconds/274 seconds for male and female).^[34]^ For lower limbs explosive force, researchers took a standing long jump test to assess it. The longer distance represents the better explosive force of the lower limbs (the cutoff of standing long jump is 208 cm and 151 cm for male and female).^[34]^ For anxiety level, it is assessed through the Zung self-rating anxiety scale (SAS). The SAS is a widespread instrument for examining anxiety disorders, while the cutoff point is 40 indicates surfer anxiety disorder.^[35]^

### 2.6. Validity and reliability of measurements

The measurement of this study involved a PT, VC, 1000/800 meter run test, standing long jump test, as well as SAS.

A repeated-measures study of 28 male and 8 female young athletes in a laboratory environment confirmed that PT is a valid, reliable, and practical method for assess core strength (for validity, it was shown by the Surface electromyography of selected core muscles, which indicated >50% increase in muscle activation during the test; for reliability, when the first attempt of 3 repeated trials was considered as familiarization, the Intraclass correlation coefficient was 0.99 (95% CI: 0.98–0.99), coefficient of variation was 2.0 ± 1.56%).^[36]^

Both VC test, 100/800 m run tests, as well as standing long jump test are the inevitable components of the annual college students physical fitness tests. This test is compulsory program hold by the Education Ministry of China. Various studies has approved the validity and reliability for those 3 measurements.^[34,37]^

As a common instrument to assess anxiety, the validity and reliability of the SAS already been detected by previous experts (Cronbach’s alpha = .82; concurrent validity *r* = 0.30).^[35]^

### 2.7. Procedure

**Figure 1.**
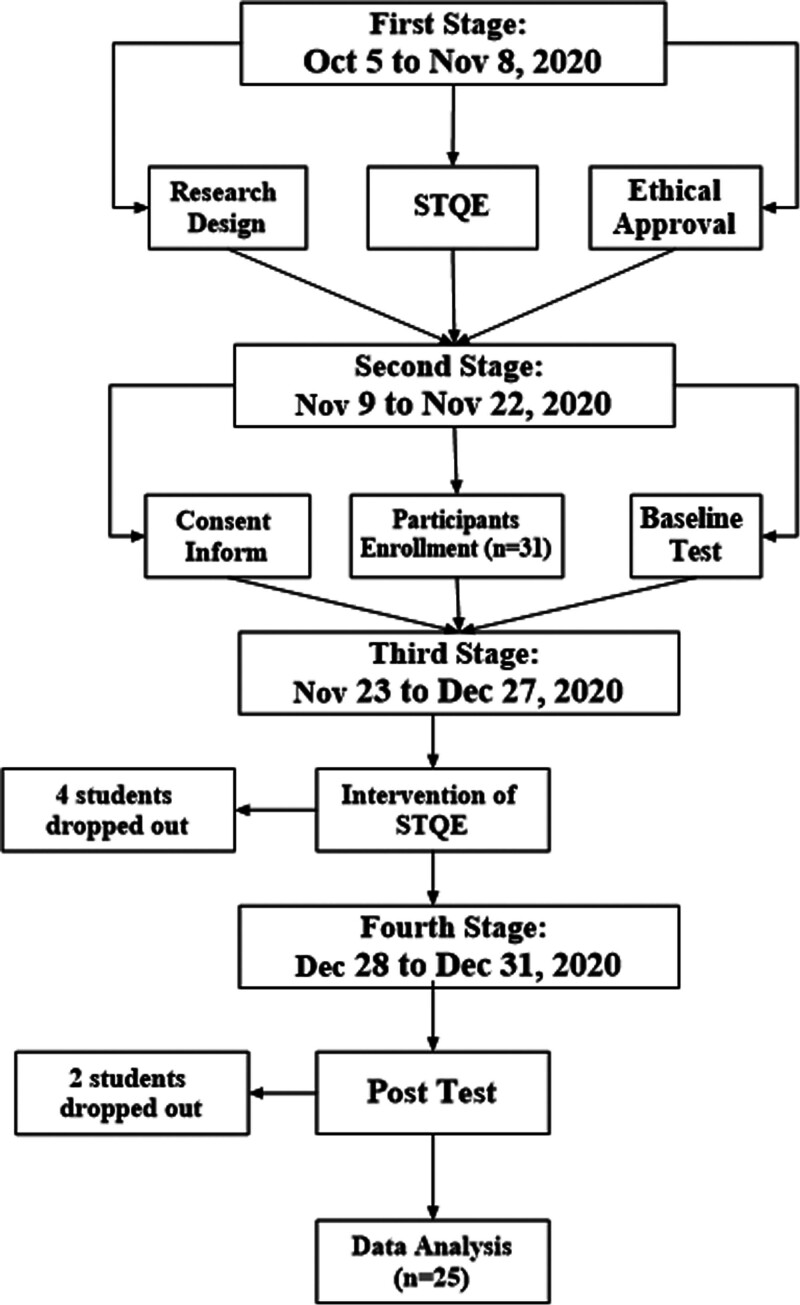
The flow chart of procedure.

The procedure of this study comprises 4 stages. In the first stage, which took place from October 5 to November 8, 2020, the authors initiated the research design, constructed the STQE, and obtained ethical approval. The second stage, spanning from November 9 to November 22, 2020, involved the enrollment of participants, the collection of consent information, and the administration of a pretest to gather baseline data. Moving on to the third stage, occurring from November 23 to December 27, 2020, a 5-week intervention of STQE was implemented. During this phase, 4 students dropped out due to frequent absenteeism. The fourth stage, from December 28 to December 31, 2020, saw the withdrawal of 2 students who failed to attend the posttest. Ultimately, 25 participants were included in the final data analysis. For a detailed overview of the specific procedure (Fig. 1).

### 2.8. Data analysis

In this study, SPSS version 21 was the instrument for data analysis. As shown in the procedure follow chart, 4 students dropped off STQE, and 2 students did not finish the posttest. Therefore, the data of 25 students would be used for analysis. The specific data methods are descriptive data and paired *t* test. The value of *P* < .05 is deemed a significant statistical difference, and *P* < .01 is deemed an extremely significant statistical difference.

## 3. Results

During this section, the research first introduces the demographic profile of participants, which is followed by the specific interpretation of the outcomes of core strength, cardiopulmonary endurance, lower limbs explosive force, and anxiety level, respectively.

### 3.1. The character of demographic

Table 1 (demographic characters) shows that a total of 25 college students in grade one finish the STQE and posttest. While 16% of participants are male, 84% are female. The average age is 18.8 ± 0.87 years old, with an average height of 161.7 ± 0.09 cm, and an average weight of 53.0 ± 12.87 kg. Moreover, their average BMI is 20.1 ± 3.58. This demographic information reveals that most participants are female students, and all of them are adolescents who have a normal BMI.

**Table 1:** Demographic characters (n = 25).

	Mean ± SD/percentage
Gender	16% male
	84% female
Age	18.8 ± 0.87
Height	161.7 ± 0.09
Weight	53.0 ± 12.87
BMI	20.1 ± 3.58

Percentage for gender, while mean and SD for others.

### 3.2. The status of baseline

According to Table 2, the baseline status of all variables was revealed. The mean and standard deviation of plan test is 110.4 ± 49.19 seconds. The mean and standard deviation of VC is 2919 ± 794.60 mL. The mean and standard deviation of RT is 276.3 ± 28.46 seconds. The mean and standard deviation of the standing jump test is 174.2 ± 25.57 cm. The mean and standard deviation of a score of Zung self-rating scale (SRS) is 42.0 ± 11.54.

**Table 2:** Comparison between baseline and posttest.

		Baseline		Posttest		Paired differences		*P*
		Mean	SD	Mean	SD	Mean	SD	
Core strength	PT	110.4	49.19	138.6	72.98	−28.1	58.71	**.025** *
Cardiopulmonary	VC	2919.0	794.60	3156.9	833.23	−237.8	766.02	.134
Endurance	RT	276.3	28.46	274.7	32.12	1.6	26.31	.764
Lower limbs								
Explosive force	SJT	174.2	25.57	180.7	27.38	−6.5	11.91	**.011** *
Anxiety level	SRS	42.0	11.54	38.6	8.42	3.4	7.83	**.039** *

PT = plank test, RT = 1000/800 m run test, SJT = standing jump test, SRS = Zung self-rating scale, VC = vital capacity.

* Indicate 0.05 level. Seconds for PT and RT, mL for VC, cm for SJT, scores for SRS.

### 3.3. The outcomes of core strength

**Figure 2.**
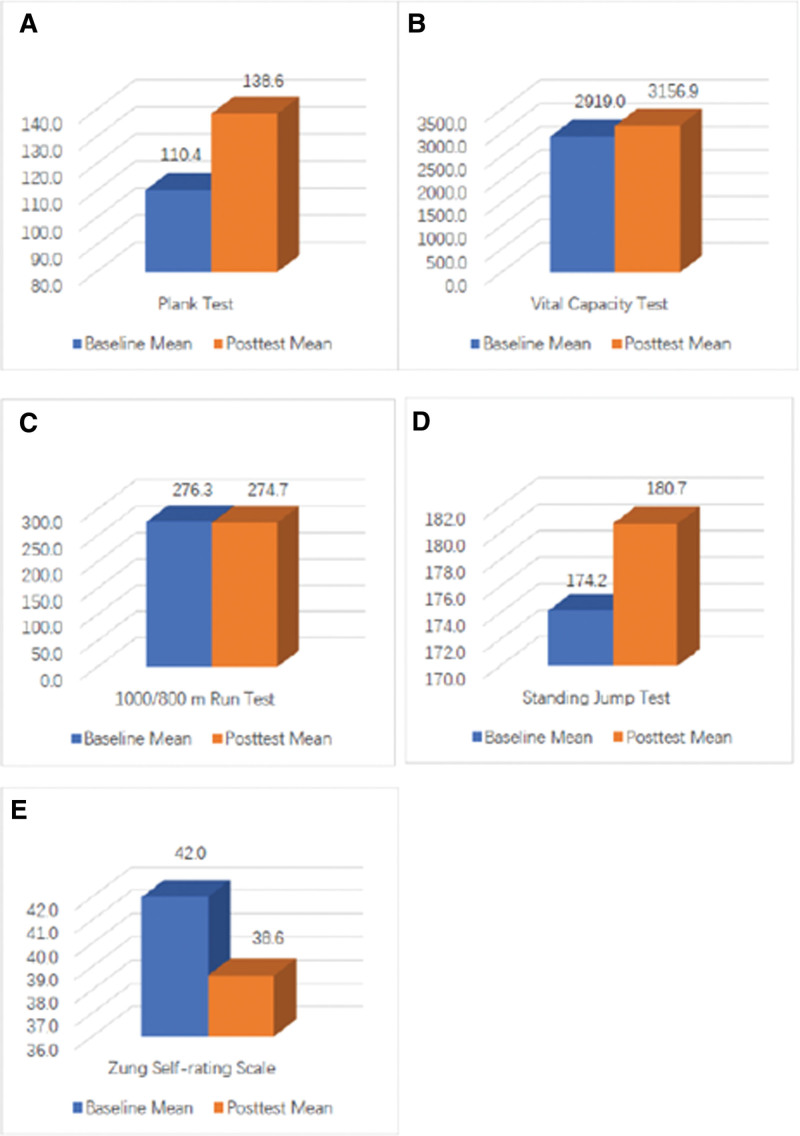
Comparison between baseline and posttest. (A) Comparison between baseline and posttest for Plank Test, (B) Comparison between baseline and posttest for Vital Capacity Test, (C) Comparison between baseline and posttest for 1000/800 m Run Test, (D) Comparison between baseline and posttest for Standing Jump Test, (E) Comparison between baseline and posttest for Zung Self-rating Scale.

According to Table 2 and Figure 2A, the mean and standard deviation of the PT have obviously increased. Specifically, it increased from 110.4 ± 49.19 to 138.6 ± 72.98, signifying an improvement of 28.1 seconds. The *P* value of the paired *t* test is 0.025, which is less than 0.05, indicating a significant statistical difference between the baseline and posttest for the PT. Therefore, STQE has a significant improvement in core strength for practitioners.

### 3.4. The outcomes of cardiopulmonary endurance

As per Table 2 and Figure 2B, there is a slight increase in the mean of VC. Specifically, it has increased from 2919 ± 794.60 to 3157 ± 833.23 mL, showing an improvement of 237.84 mL. However, the *P* value of the paired *t* test is 0.134, which is greater than 0.05, indicating there is no significant statistical difference between the baseline and posttest for VC. Referring to Table 2 and Figure 2C, there is barely any change in the mean of the RT. Specifically, the posttest shows a reduction of only 1.6 seconds. Moreover, the *P* value of the paired *t* test is 0.764, which is greater than 0.05, suggesting there is no significant statistical difference between the baseline and posttest for the RT. Therefore, the STQE does not significantly impact the cardiopulmonary endurance of college students.

### 3.5. The outcomes of lower limbs explosive force

As evidenced by Table 2 and Figure 2D, there is a clear increase in the mean and standard deviation of the standing long jump test. Specifically, it has increased from 174 ± 25.57 to 181 ± 27.38 cm, an improvement of 6.52 cm. The *P* value from the paired *t* test is 0.011, which is less than .05, indicating that there is a significant statistical difference between the baseline and posttest for the lower limbs explosive test. Therefore, the STQE has a significantly positive effect on improving the lower limbs’ explosive force of individuals.

### 3.6. The outcomes of anxiety level

As displayed in Table 2 and Figure 2E, while the mean scores of the PT and standing jump test exhibit a considerable increase, the score of the SRS notably declines in the posttest. Specifically, the SRS scores decrease from 42 ± 11.54 to 38.6 ± 8.42, resulting in a reduction of 3.41. The *P* value from the paired *t* test is 0.039 < 0.05, indicating a significant statistical difference between the baseline and posttest for the anxiety level, not the lower limb explosive test. Therefore, it can be concluded that the STQE has a significant effect on reducing students’ anxiety levels.

## 4. Discussion

The results indicate a considerable increase in the core strength of university students following STQE (28.1 seconds in the PT, *P* = .025). Some reasons may be attributed to it. STQE is a new mind-body exercise training which combines Tai Chi and Qigong together. Tai Chi and Qigong exercises have to stress the spine, waist, as well as abdomen.^[38–40]^ Tai Chi consists of various movements with waist and abdominal stretching; it is exactly what core training does through other difficult methods like ball workouts with the waist.^[41]^ Qigong emphasizes conducting qi to Dantian (an acupoint). It means gathering inner energy in the lower abdomen.^[42,43]^ Another important factor is abdominal breathing in Qigong and Tai Chi. Abdominal breathing, also named Dantian respiration, requires slow and deep breathing with a shrinking and extensive belly.^[44]^ This waist stretching exercise conducted qi to Dantian and abdominal breathing in STQE, can improve abdominal muscle strength and waist power, which is vitally important to core strength.^[8,45]^ Previous studies also show that Qigong can significantly improve core stability.^[46]^ Therefore, STQE can significantly improve practitioners’ core strengths.

Contrary to core strength, our research shows that STQE does not significantly improve an individual’s cardiopulmonary endurance. There is only a slight increase (an increase of 237.84 mL; *P* = .134) in VC and almost no change (reduction of 0.01 seconds; *P* = .923) in the RT. Environmental factors like seasons and temperature may have contributed to this. The pretest was administered in the middle of November, and the posttest was administered at the end of December. At Nanning, the average temperature in November is 19.1 °C, the average temperature in December is 14.3 °C, and the minimum temperature in December is 10.3 °C.^[47]^ Residents’ cardiopulmonary endurance may be reduced as the ambient temperature drops. Galan-Carracedo and his team supported this opinion. They deem that environmental temperature determines skin temperature during exercise, and skin temperature has a positive connection to cardiopulmonary endurance.^[48]^ Another reason is the type of exercise. STQE combines Tai Chi and Qigong exercises together. It is still a moderate-intensity exercise. However, compared with high-intensity interval training, moderate intensity has a slight influence on cardiorespiratory fitness.^[49]^ Moreover, the previous study confirmed that the improvements in cardiopulmonary function occurred after 12 and 16 weeks of exercise interventions, rather than in the 8-week.^[50]^ However, the STQE only involved 5 weeks. Consequently, STQE failed to enhance individuals’ heart and lung function. In other words, the short exercise of Taichi and qigong is not efficacy for cardiopulmonary fitness.

Furthermore, results indicate that STQE significantly increases (an increase of 6.52 cm; *P* = .011) the lower limbs explosive force among university students. This could be due to the crouched position maintained throughout the STQE training process, which puts a certain load on the muscles of the lower limbs, improving the strength of the knee extensor and flexor muscles.^[51]^ The second factor is relaxation. Relaxation is the key principle for Qigong exercise and Tai Chi.^[52]^ The relaxation helps to improve the extensive muscle agility of the brain nerve, which is critical for explosive power.^[53]^ Both the crouched position and the relaxation of STQE stimulate the strength of the lower limb muscles. The results are consistent with previous studies that found Tai Chi can improve individuals’ lower limb strength.^[54]^ Thus, STQE can dramatically enhance individuals’ lower limbs explosive force.

Besides, these results also indicated that STQE has a clear reduce in the anxiety level of university students. Abdominal breathing and relaxation in STQE may also contribute to it. Abdominal breathing is an effective way to relax the mental state of residents.^[55]^ This kind of slow and deep breathing can regulate the brain’s emotions via altering the autonomic system and restoring homeostasis and the modulation of autonomic nervous functions.^[56,57]^ Abdominal breathing triggers body relaxation responses and benefit a person’s mental health by reducing negative affect and enforcing emotional improvement.^[58]^ Moreover, Chen and his team point out that abdominal breathing and relaxation training can achieve significant reductions in Beck Anxiety Inventory scores.^[59]^ Besides, Henz’s team highlighted that Qigong exercise can increase alpha activity, which leads to a relaxed state of mind to alleviate anxiety symptoms.^[52]^ Therefore, STQE can diminish students’ anxiety levels in this study. This is consistent with various previous studies which found Tai Chi^[60–62]^ or Qigong^[63,64]^ can benefit residents’ mental health.

In summary, STQE has significantly improved core strength and lower limbs explosive force and an obviously reduced anxiety level. But STQE has no significant effects on cardiopulmonary endurance. Therefore, this study rejects Hypothesis 1 (STQE has a significant improvement on university students’ core strength, cardiopulmonary endurance, and low limb explosive force) and accepts Hypothesis 2 (STQE has a significant reduction on university students’ anxiety level).

The main significance of this study is to bridge the practical knowledge gap between the long period of conventional Tai Chi Qigong program and the fast-paced society. This study provides an appropriate short Taichi Qigong program for humans in a modern lifestyle. Moreover, our research also provides practical evidence for Qigong’s contribution to cardiopulmonary endurance, which is still unclear in past research.^[65]^

## 5. Limitations

Inevitably, there are some limitations to this study. First, there is no control group, so the researcher cannot compare it with conventional Tai Chi and Qigong programs. Second, the instrument for dependent variables is easy and primary, particularly in cardiopulmonary endurance. Just take VC and a RT. This may cause some bias in the results. Thirdly, there are some shortcomings in the research design such as lack of lack of blinding and follow up study. Finally, not mentioned the difference in gender.

Therefore, to further research, scholars can add a control group, blinding, follow up study, gender difference, and use more precise instruments for dependent variables.

## 6. Conclusions

This study revealed that STQE has an obviously positive effect on core strength, lower limbs explosive force, as well as anxiety level for university students. However, STQE has no significant effects on university students’ cardiopulmonary endurance. Additionally, this results may be weakened due to the research's non-randomized design and with limitations as above statement.

## Author contributions

**Conceptualization:** Feng Wang.

**Data curation:** Feng Wang, Syed Kamaruzaman Bin Syed Ali.

**Investigation:** Feng Wang.

**Methodology:** Feng Wang, Syed Kamaruzaman Bin Syed Ali.

**Supervision:** Syed Kamaruzaman Bin Syed Ali.

**Validation:** Feng Wang, Syed Kamaruzaman Bin Syed Ali.

**Visualization:** Feng Wang.

**Writing – original draft:** Feng Wang.

**Writing – review & editing:** Feng Wang.
